# Timing luteal urinary pregnanediol glucuronide assessment using urinary luteinizing hormone: agreement with ultrasound-determined ovulation

**DOI:** 10.3389/fendo.2026.1992715

**Published:** 2026-10-12

**Authors:** Rene Leiva, Rene Ecochard, Jonathan Yang

**Affiliations:** 1 Department of Family Medicine, Faculty of Medicine, University of Ottawa, Ottawa, ON, Canada; 2 Bruyère Health Research Institute, Ottawa, ON, Canada; 3 Hospices Civils de Lyon, Service de Biostatistique-Bioinformatique, Lyon, France; 4 Centre National de la Recherche Scientifique (CNRS), Laboratoire de Biométrie et Biologie Évolutive, Équipe Biostatistique-Santé, Villeurbanne, France; 5 Department of Family Practice, Faculty of Medicine, University of British Columbia, Vancouver, BC, Canada

**Keywords:** fertility awareness, luteal phase, luteal progesterone exposure, mid-luteal phase, progesterone metabolite, urinary hormone monitoring

## Abstract

**Background:**

Urinary pregnanediol glucuronide (PdG) is a non-invasive marker of luteal progesterone exposure, but its interpretation depends on timing relative to ovulation. We evaluated whether urinary luteinizing hormone (LH)-derived reference points could approximate ultrasound-determined ovulation (USDO) for scheduling mid-luteal PdG assessment.

**Methods:**

This retrospective secondary analysis included 283 ultrasound-confirmed ovulatory cycles from 102 women. We evaluated the retrospectively identified urinary LH peak and the first urinary LH concentrations ≥10, ≥15, ≥20, ≥25, and ≥30 mIU/mL. PdG measured 6–9 days after each LH reference was compared with PdG measured on the corresponding USDO-timed day. A PdG threshold of ≥15 µg/mL was used for the primary descriptive classification, with sensitivity analyses at ≥5, ≥10, and ≥20 µg/mL. Ninety-five percent confidence intervals were estimated using participant-level cluster bootstrap.

**Results:**

First urinary LH ≥15 mIU/mL occurred a median of 0 days from USDO (IQR −0.5 to +1), with 70.0% and 80.7% of events occurring within ±1 and ±2 days, respectively. At PdG ≥15 µg/mL, overall agreement ranged from 74.3% to 82.2% across days +6 to +9, with AUC values of 0.764–0.835. Alternative PdG thresholds produced different trade-offs between positive and negative agreement, while ≥15 µg/mL provided a comparatively balanced descriptive profile.

**Conclusions:**

First urinary LH ≥15 mIU/mL is a candidate prospective reference for scheduling day-specific mid-luteal urinary PdG assessment. The present study establishes a candidate timing strategy rather than a clinically actionable decision rule; a single LH-timed PdG result should not be used alone to diagnose luteal dysfunction or direct treatment. These findings are hypothesis-generating; numerical LH and PdG thresholds are assay-dependent, require prospective platform-specific validation, and should not be interpreted as diagnostic criteria for luteal phase deficiency unless validated in a clinical trial.

## Highlights

- Day-specific analyses compared PdG measured at LH + 6 through LH + 9 directly with PdG measured on the corresponding USDO + 6 through USDO + 9 days.
- First urinary LH ≥15 mIU/mL was centered on USDO and was identified in more evaluable cycles than higher prospective LH concentration thresholds.
- Sensitivity analyses using PdG thresholds of ≥5, ≥10, ≥15, and ≥20 µg/mL demonstrated threshold-dependent trade-offs; ≥15 µg/mL provided a comparatively balanced descriptive agreement profile.
- The findings are hypothesis-generating, and the numerical LH and PdG thresholds require prospective, platform-specific validation.

## Introduction

Appropriate progesterone exposure during the luteal phase is essential for secretory transformation of the endometrium and the establishment of endometrial receptivity (1, 2). Consequently, accurate assessment of luteal progesterone production has become increasingly relevant in fertility awareness, infertility evaluation, assisted reproduction, and home fertility monitoring (3–7). Although serum progesterone remains the most commonly used biochemical marker of luteal function, its clinical interpretation is limited by marked pulsatile secretion and substantial intra-day variability (8). A single serum progesterone measurement therefore provides only a brief snapshot of ovarian steroid production and may not accurately reflect overall luteal progesterone exposure.

Urinary pregnanediol glucuronide (PdG), the principal urinary metabolite of progesterone, provides an attractive alternative for assessing luteal progesterone production (9). Because PdG reflects progesterone metabolism over the urine collection interval rather than a single moment in time, first-morning urinary measurements offer a practical, non-invasive method for repeated assessment in both clinical practice and home settings (9, 10). Advances in quantitative urinary hormone assays and smartphone-assisted fertility monitoring have renewed interest in urinary PdG as a biomarker of ovulation confirmation and luteal function (11–14). Previous studies have described physiological urinary PdG profiles referenced to independently determined ovulation and have demonstrated a characteristic post-ovulatory rise followed by sustained mid-luteal concentrations (10, 14, 15). These observations have established urinary PdG as a biologically plausible marker of luteal progesterone exposure, but its clinical usefulness depends critically on appropriate timing of measurement (16, 17).

In research settings, the timing of ovulation is most accurately determined using serial transvaginal ultrasonography, which allows urinary hormone measurements to be aligned precisely with follicular rupture (18). Ultrasound-based timing has therefore served as the reference method for describing periovulatory hormone profiles and evaluating luteal endocrine physiology. However, serial ultrasonography is expensive, labor-intensive, and impractical for routine clinical care or home fertility monitoring. Outside research environments, clinicians and patients typically estimate ovulation using urinary luteinizing hormone (LH) rather than ultrasound (19). Recent longitudinal evidence demonstrating substantial within-woman and between-woman variability in ovulation timing across repeated menstrual cycles underscores the limitations of fixed calendar-based assessment and supports the evaluation of cycle-specific physiological events, such as urinary LH, as individualized temporal references (20).

Urinary LH testing is widely available and forms the basis of most contemporary fertility-monitoring systems. Nevertheless, uncertainty remains regarding which LH-derived reference point should be used to schedule subsequent PdG assessment. The urinary LH surge varies considerably among menstrual cycles with respect to onset, duration, magnitude, and peak concentration (21, 22). Multiple or atypical LH elevations can further complicate interpretation, and some cycles do not reach higher quantitative LH thresholds. Consequently, different LH-based timing strategies may lead to materially different PdG sampling days and, therefore, different interpretations. These limitations provide the rationale for evaluating several quantitative LH reference definitions rather than assuming a single universal surge threshold.

Several commercially available fertility-monitoring systems now combine urinary LH and PdG measurements to identify ovulation and characterize the luteal phase (12). However, recommendations regarding when PdG should be measured after an LH event are largely based on expert opinion, manufacturer guidance, or limited physiological data rather than direct comparison with ultrasound-confirmed ovulation. Furthermore, previous investigations have generally focused on confirming ovulation or describing normative hormone patterns rather than evaluating whether clinically practical LH-based strategies can reproduce ultrasound-referenced timing for PdG assessment.

The present study addressed a methodological question rather than evaluating luteal phase deficiency, implantation competence, or pregnancy outcomes. Using daily quantitative urinary hormone measurements and ultrasound-confirmed ovulation, we compared several clinically relevant LH-derived reference points, including the retrospectively identified urinary LH peak and prospectively identifiable urinary LH concentration thresholds, for timing urinary PdG assessment during the mid-luteal interval. This interval was selected because it overlaps the period during which luteal progesterone exposure is physiologically sustained and during which implantation is expected to occur. Lessey estimated that endometrial receptivity spans approximately 3–6 days of the secretory phase (23); Muter et al. reported that receptivity begins approximately 6 days after the LH surge and persists for about 4 days (24); and Wilcox et al. observed that in natural conceptions implantation occurs predominantly 8–10 days after ovulation (25). These observations motivate the choice of a mid-luteal assessment interval, but they are not endpoints of the present study: no implantation, endometrial receptivity, pregnancy, or other reproductive outcome was evaluated here, and the interval should be read as a mid-luteal sampling interval rather than as a validated window of implantation. We therefore hypothesized that one or more LH-based timing strategies would demonstrate high agreement with ultrasound-guided timing while remaining practical for routine clinical use.

The primary objective was to evaluate whether urinary LH-derived reference points could provide a practical substitute for ultrasound when scheduling mid-luteal urinary PdG assessment. We assessed day-specific agreement by comparing PdG measured 6–9 days after each LH reference point with PdG measured on the corresponding days after USDO. Secondary objectives were to compare LH concentration thresholds, characterize their temporal relationship to USDO, and identify a practical prospective strategy when ultrasound is unavailable.

## Materials and methods

### Study design

This was a retrospective secondary analysis of prospectively collected observational menstrual-cycle data. The present analysis evaluated whether clinically applicable urinary luteinizing hormone (LH)-based timing strategies could substitute for ultrasound-based timing of urinary pregnanediol glucuronide (PdG) assessment. The original study was approved by the Comité Consultatif de Protection des Personnes dans la Recherche Biomédicale de Lyon. All participants provided written informed consent, and study procedures were conducted in accordance with the Declaration of Helsinki (18). The present study was a secondary analysis of previously collected data from the original study. No additional participant recruitment, intervention, specimen collection, or data collection was undertaken for the present analysis, and no separate ethics review was sought for this secondary analysis.

### Participants

Participants were recruited from 1996 to 1997 at eight natural family planning clinics in France, Italy, Germany, Belgium, and Spain. The original study enrolled 107 healthy reproductive-aged women contributing 326 cycles. For the present analyses, cycles were eligible when ultrasound-confirmed ovulation and the hormone measurements required for the relevant comparison were available. Eligibility was not conditioned on the observed agreement between LH and USDO. Of the 326 cycles in the original cohort, 283 cycles from 102 women had ultrasound-confirmed ovulation and formed the analytical cohort for the primary analyses before analysis-specific missingness. Cycles were not evaluable if ultrasound did not confirm a day of ovulation (n=43), or when the urinary LH or PdG measurements required for the relevant comparison were unavailable across the assessment window; the corresponding cycle- and participant-level counts are shown in **Figure 1**.

**Figure 1:**
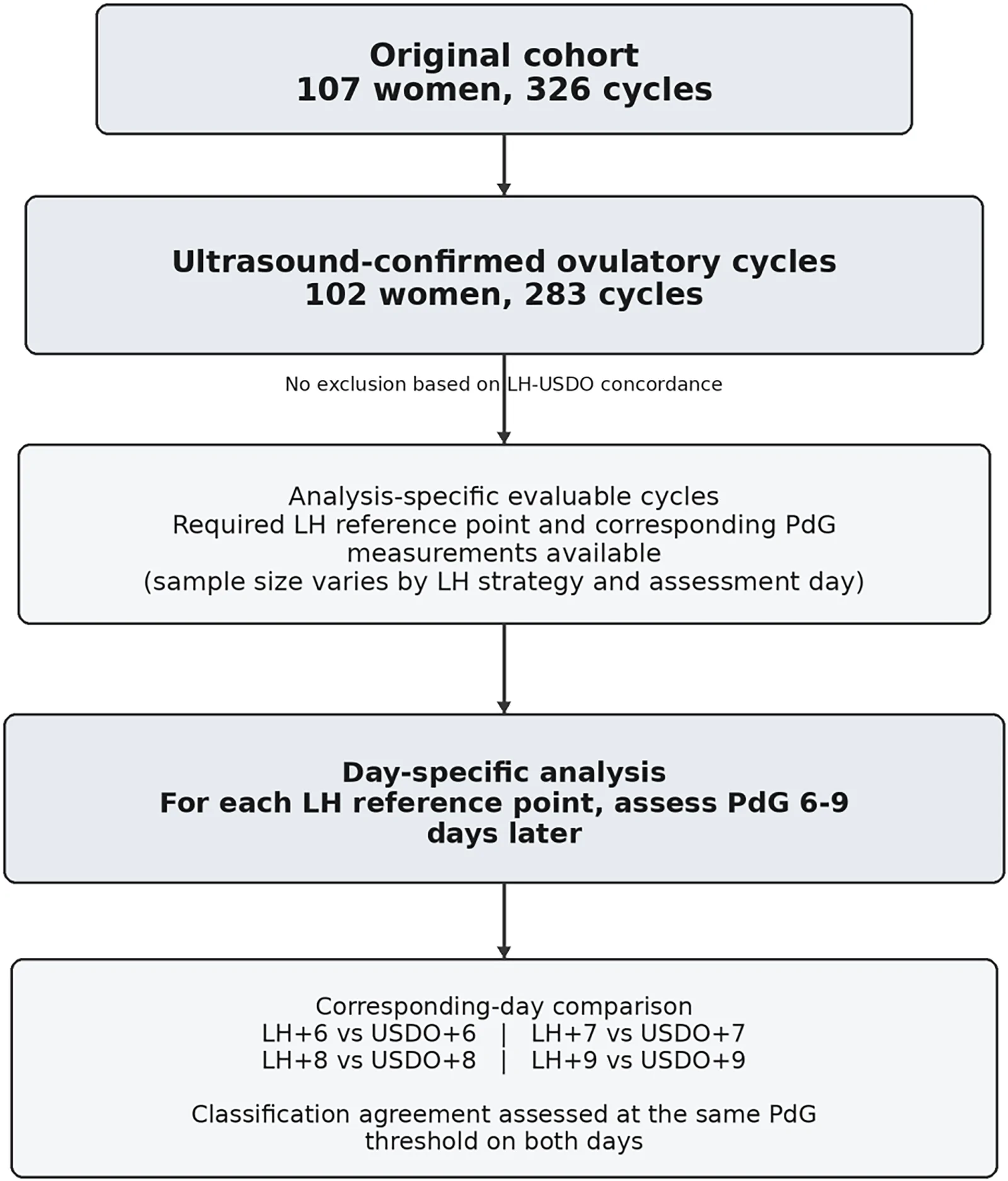
Flow of participants and menstrual cycles in the day-specific analyses. The original cohort included 107 women contributing 326 cycles; 283 ultrasound-confirmed ovulatory cycles from 102 women formed the unrestricted analytical cohort before analysis-specific missingness. No cycle was excluded on the basis of LH-USDO concordance. Evaluable sample sizes varied by LH strategy and assessment day according to availability of the required LH reference point and PdG measurement. LH-timed PdG at +6, +7, +8, and +9 days was compared with PdG on the corresponding USDO-timed day.

### Urinary hormone measurements

Early morning urine samples were collected daily, and a 10–12 mL aliquot was transferred into a tube containing gentamicin sulphate. The tubes were frozen at −20 °C until analysis. Frozen urine specimens were assayed for quantitative detection of pregnanediol-3α-glucuronide (PdG) and luteinizing hormone using time-resolved fluorometric immunosorbent assays (DELFIA). All samples from a single participant were tested in duplicate in the same assay. Numerical inter-assay coefficients of variation are not available in the archived study records and therefore cannot be reported. The archived records indicate that specimens were thawed once before analysis; however, the available documentation does not allow us to determine whether samples from all participants were analyzed in a single analytical session or across multiple analytical sessions. Intra-assay variation, calculated as the relative difference |value 1 − value 2|/mean value, was 5.7% for PdG and 7.9% for LH. The present analyses use the original historical measurements generated in the source study; no new analytical determinations were produced for this secondary analysis. Hormone concentrations were analyzed as reported, without creatinine normalization, and all decision thresholds in this manuscript therefore apply to uncorrected urinary concentrations (PdG, µg/mL; LH, mIU/mL). Further analytical performance characteristics of the historical assays — reagent manufacturer, calibrator material, calibration range, limits of detection and quantification, and cross-reactivity data — are not retrievable from the original study records and are therefore not reported. This limits the analytical traceability of the specific numerical thresholds proposed here, and is considered further in the Limitations.

### LH timing strategies

Two categories of urinary LH timing strategies were evaluated. The LH peak was calculated separately for each menstrual cycle using daily quantitative urinary LH measurements and was defined as the day with the highest urinary LH concentration; when multiple days shared the same maximum value, the earliest occurrence was selected. Prospectively identifiable LH thresholds were defined as the first day on which urinary LH reached or exceeded 10, 15, 20, 25, or 30 mIU/mL. These thresholds span the ascending and peak phases of the urinary LH surge (**Figure 2**). In this analysis, no cycle was excluded because of the observed LH-USDO timing difference.

**Figure 2:**
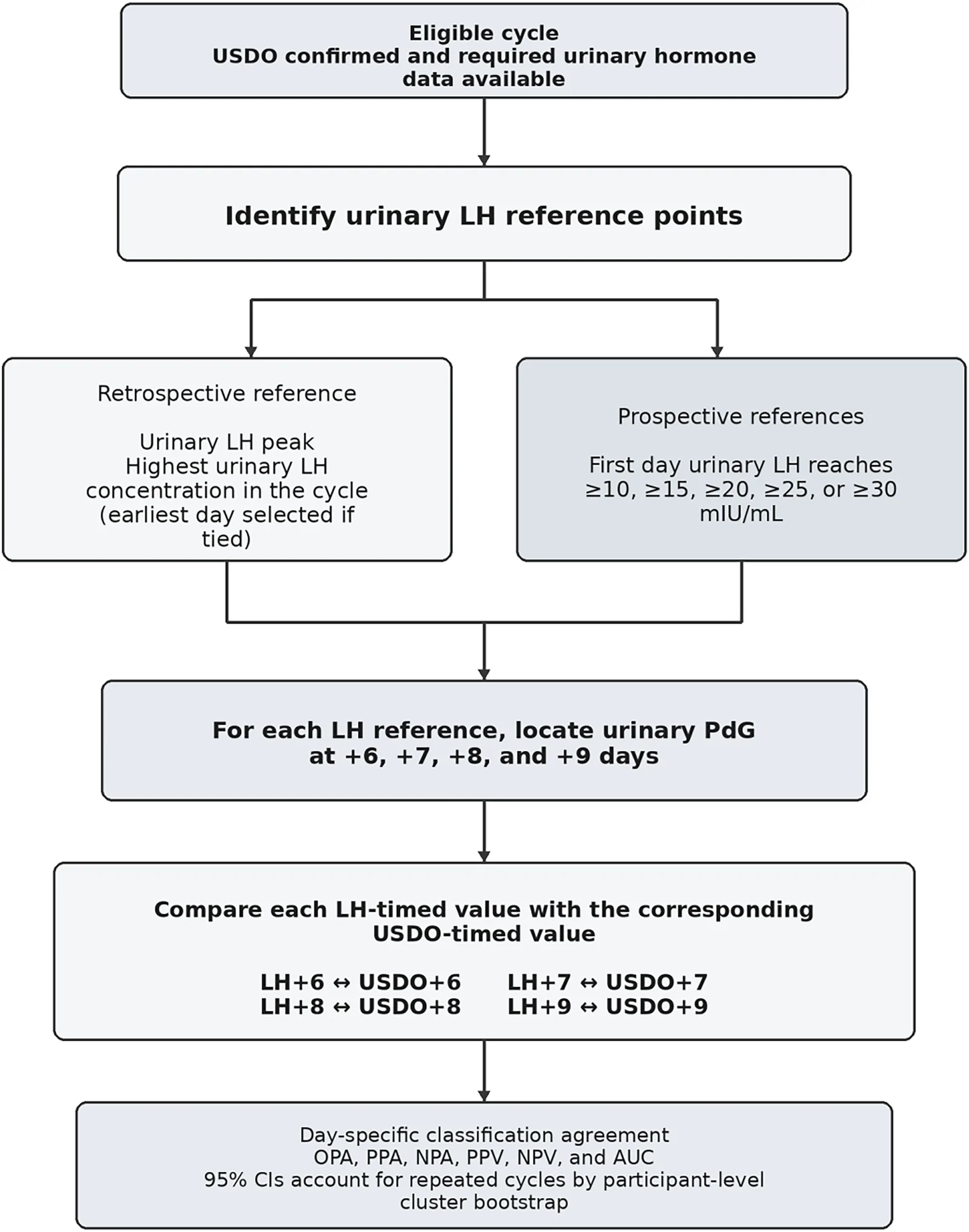
Algorithm for identification of urinary LH reference points and day-specific PdG comparison. The retrospective reference was the urinary LH peak (earliest day selected if tied); prospective references were the first days urinary LH reached ≥10, ≥15, ≥20, ≥25, or ≥30 mIU/mL. For each reference, PdG at LH + 6 through LH + 9 was compared with PdG on the corresponding USDO + 6 through USDO + 9 day.

### Sensitivity analysis of the PdG classification threshold

To assess whether classification agreement was dependent on the selected urinary PdG threshold, the complete day-specific analysis was repeated using PdG cutoffs of ≥5, ≥10, ≥15, and ≥20 µg/mL. For each cutoff, all six LH reference strategies—the retrospectively identified LH peak and the first urinary LH concentrations ≥10, ≥15, ≥20, ≥25, and ≥30 mIU/mL—were evaluated at assessment days +6, +7, +8, and +9. Each LH-timed PdG measurement was compared with the PdG measurement obtained on the corresponding USDO-timed day (e.g., LH + 7 versus USDO + 7), with the same PdG threshold applied to both measurements. For a given LH strategy and assessment day, the evaluable cycle set was unchanged across PdG thresholds; changing the cutoff therefore altered classification status but not analytic eligibility. For each combination, 2×2 classification counts (true positive, false positive, true negative, and false negative) were generated, and overall percent agreement (OPA), positive percent agreement (PPA), negative percent agreement (NPA), positive predictive value (PPV), negative predictive value (NPV), and area under the receiver operating characteristic curve (AUC) were calculated. Ninety-five percent confidence intervals were estimated using participant-level cluster bootstrap to account for repeated cycles contributed by the same woman.

### Ultrasound determination of ovulation

As previously described (18, 26), transvaginal ultrasonography was used to monitor each ovulation. This was to be performed since the onset of fertile type cervical mucus and/or luteinizing hormone surge as detected by urinary rapid assays provided by the original researchers. Daily, subjects were to perform two rapid assays on morning urine: the Bluetest Ovulation test (Quidel) and the luteinizing hormone Conceive test (Quidel). Scanning was first performed on each other day until observation of 16-mm follicles, then daily until evidence of ovulation. The day of ovulation (USDO) was defined as the 24-hour time-lag between the visualization of a mature follicle at one scan and the appearance of a ruptured follicle or an early corpus luteum, and/or free fluid in the cul-de-sac at the next scan. Precisely, ultrasound signs of luteinization and/or follicle rupture were either a disappearance or a change in size, shape, or sonographic density. The ultrasound-determined day of ovulation (USDO) was designated Day 0, with days before and after ovulation expressed relative to this reference (26).

### Statistical analysis

Participant characteristics and daily USDO-aligned PdG concentrations were summarized descriptively. The unit of analysis was the menstrual cycle. For each LH and PdG strategies and assessment day, we calculated the 2×2 classification table and overall percent agreement (OPA), positive percent agreement (PPA), negative percent agreement (NPA), positive predictive value (PPV), negative predictive value (NPV), and the area under the receiver operating characteristic curve (AUC), using the continuous LH-timed PdG concentration for the AUC. No formal inferential comparison among the +6, +7, +8, and +9 assessment days, or among the competing LH thresholds, was prespecified; differences among strategies and days were therefore interpreted descriptively. Because some women contributed more than one menstrual cycle, 95% confidence intervals were estimated by participant-level cluster bootstrap, resampling women with replacement and retaining all eligible cycles contributed by each sampled woman. This approach preserves within-woman dependence without changing the cycle as the unit of analysis. Timing differences between each LH reference event and USDO were also summarized by the median, interquartile range, and proportions within ±1 and ±2 days. Analyses were performed using R (version 4.4.1; R Foundation for Statistical Computing, Vienna, Austria).

## Results

### Participant characteristics

The original study enrolled 107 women contributing 326 menstrual cycles. The primary unrestricted analysis identified 283 ultrasound-confirmed ovulatory cycles from 102 women before analysis-specific missingness (**Figure 1**). The number of evaluable cycles varied by LH strategy and assessment day according to availability of the required LH and PdG measurements. Baseline characteristics of the 102 women are summarized in **Table 1**. The exact number of ultrasound examinations performed per cycle is not available in the archived study records. However, 152 cycles included at least two ultrasound examinations, of which 69 included more than two. The 102 women contributed a median of 3 cycles each: 9 women contributed 1 cycle, 15 contributed 2 cycles, 68 contributed 3 cycles, and 10 contributed 4 cycles.

**Table 1:** Baseline characteristics of the participants in the primary unrestricted cohort (N = 102).

Variable	Mean ± SD or n (%)
Age (years)	32.4 ± 5.8
Weight (kg)	57.5 ± 8.0
Height (cm)	164.4 ± 6.7
Age at menarche (years)	13.2 ± 1.6
Number of children	1.8 ± 1.7
Vegetarian	4 (3.9)
Current smoker	11 (10.8)

Values are mean ± standard deviation for continuous variables and n (%) for categorical variables.

### Hormone profile

Daily urinary PdG concentrations demonstrated the expected physiological pattern when aligned to the ultrasound-determined day of ovulation (**Figure 3**). Median PdG concentrations remained low throughout the follicular phase, increased rapidly after ovulation, and reached sustained mid-luteal concentrations before declining during the late luteal phase. Variability between participants increased after ovulation, although the overall temporal pattern remained consistent across menstrual cycles. The complete day-by-day urinary PdG reference values relative to USDO are provided in **Table 2**.

**Figure 3:**
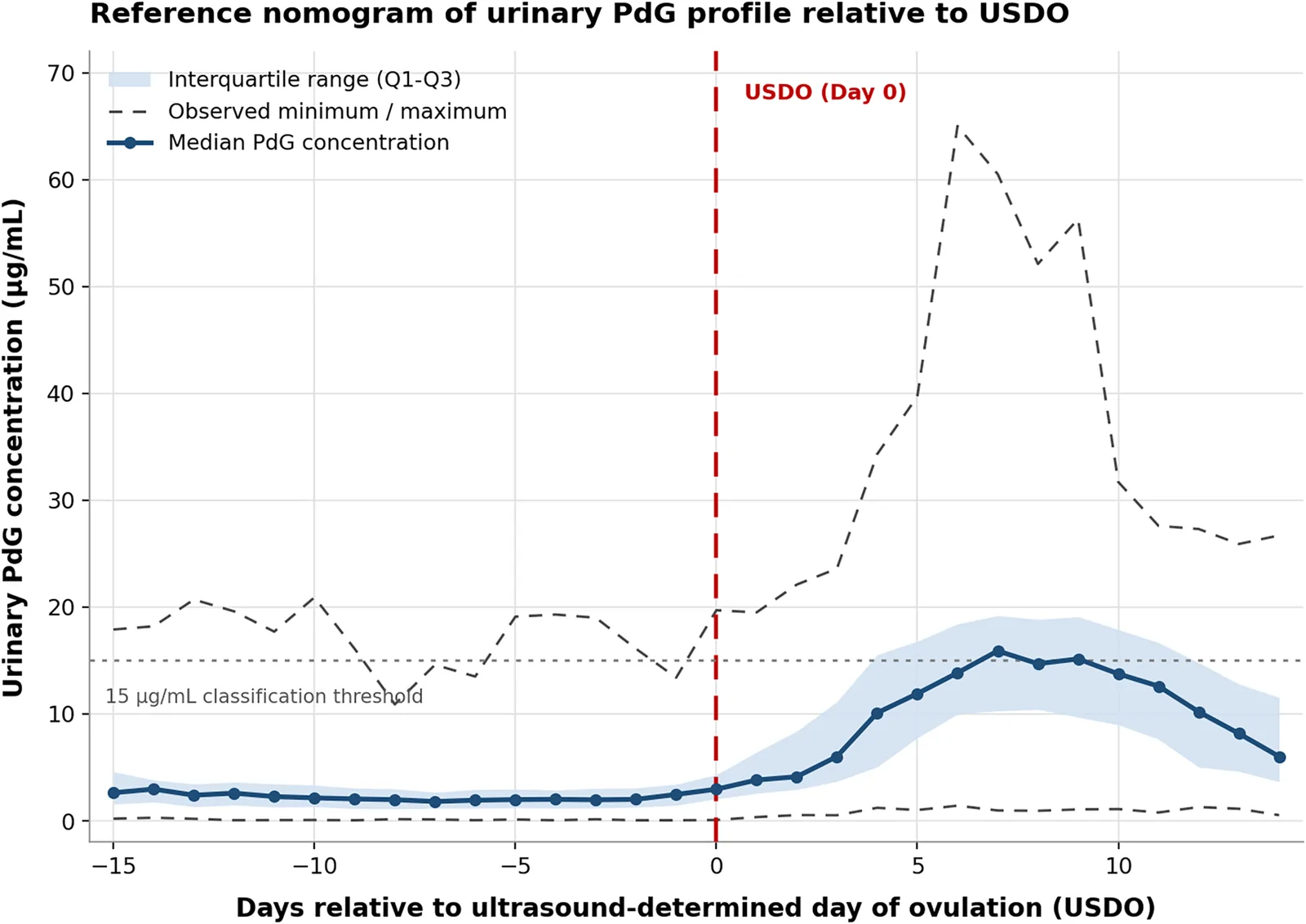
Reference nomogram of urinary pregnanediol glucuronide (PdG) concentrations relative to the ultrasound-determined day of ovulation (USDO). Caption: The solid line represents the median urinary PdG concentration and the shaded band the interquartile range (Q1–Q3); dashed grey lines indicate the observed minimum and maximum values. The vertical red dashed line denotes USDO (Day 0) and the horizontal dotted line the 15 µg/mL classification threshold. PdG concentrations are reported in µg/mL.

**Table 2:** Daily urinary pregnanediol glucuronide (PdG) concentrations relative to the ultrasound-determined day of ovulation (USDO).

Day relative to USDO	Median PdG (µg/mL)	First quartile Q1 (µg/mL)	Third quartile Q3 (µg/mL)	Range, min–max (µg/mL)
−15	2.63	1.58	4.59	0.20–17.90
−14	2.97	1.76	3.83	0.29–18.20
−13	2.40	1.33	3.45	0.19–20.70
−12	2.59	1.48	3.63	0.06–19.60
−11	2.27	1.30	3.46	0.07–17.70
−10	2.15	1.33	3.34	0.08–20.90
−9	2.05	1.15	3.07	0.05–16.10
−8	1.98	1.13	2.99	0.16–10.90
−7	1.81	1.18	2.68	0.13–14.60
−6	1.93	1.19	2.94	0.06–13.50
−5	1.99	1.22	2.98	0.13–19.10
−4	2.01	1.19	2.89	0.05–19.30
−3	1.97	1.21	3.04	0.15–19.00
−2	2.01	1.28	3.09	0.05–16.10
−1	2.47	1.46	3.43	0.05–13.40
0	2.96	2.06	4.32	0.08–19.70
+1	3.82	2.58	6.44	0.35–19.50
+2	4.13	2.94	8.39	0.54–22.10
+3	6.02	3.70	11.15	0.52–23.60
+4	10.10	5.05	15.55	1.22–34.30
+5	11.90	7.77	16.80	1.01–39.70
+6	13.85	10.00	18.43	1.42–65.00
+7	15.90	10.30	19.20	0.97–60.50
+8	14.70	10.43	18.85	0.94–52.10
+9	15.15	9.69	19.10	1.07–56.30
+10	13.75	8.99	17.90	1.10–31.70
+11	12.60	7.64	16.68	0.78–27.60
+12	10.20	5.03	14.80	1.29–27.30
+13	8.17	4.63	12.80	1.13–25.90
+14	6.02	3.67	11.55	0.53–26.70

Values are expressed as median, first quartile (Q1), third quartile (Q3), and observed range. Day 0 represents the ultrasound-determined day of ovulation (USDO). PdG concentrations are reported in µg/mL.

### Timing of LH reference points relative to USDO

The timing of each urinary LH reference point relative to USDO is summarized in **Table 3**. The first urinary LH ≥15 mIU/mL was centered on USDO (median difference 0 days; IQR −0.5 to +1), with 70.0% of reference points within ±1 day and 80.7% within ±2 days. Higher thresholds were similarly centered but reduced cycle availability: 215 cycles reached ≥20 mIU/mL, 191 reached ≥25 mIU/mL, and 164 reached ≥30 mIU/mL, compared with 243 cycles reaching ≥15 mIU/mL. The retrospectively identified LH peak occurred at a median of +1 day relative to USDO and was available in 283 cycles. The distributions of timing differences for all urinary LH reference points are shown in **Figure 4**.

**Table 3:** Timing of urinary LH reference points relative to the ultrasound-determined day of ovulation (USDO).

LH reference point	Evaluable cycles (n)	Median difference (days)	IQR (days)	Within ±1 day (%)	Within ±2 days (%)
LH peak	283	+1	0 to +2	55.8	76.7
First LH ≥10 mIU/mL	267	0	−3 to 0	58.8	70.8
First LH ≥15 mIU/mL	243	0	−0.5 to +1	70.0	80.7
First LH ≥20 mIU/mL	215	0	0 to +1	70.2	83.3
First LH ≥25 mIU/mL	191	0	0 to +1	69.6	84.8
First LH ≥30 mIU/mL	164	+1	0 to +2	69.5	84.1

The timing difference is the LH reference day minus USDO, in days; negative values indicate that the LH reference event occurred before USDO. Evaluable cycles are those in which the relevant LH reference point was identified. The corresponding distributions are shown in **Figure 4**.

**Figure 4:**
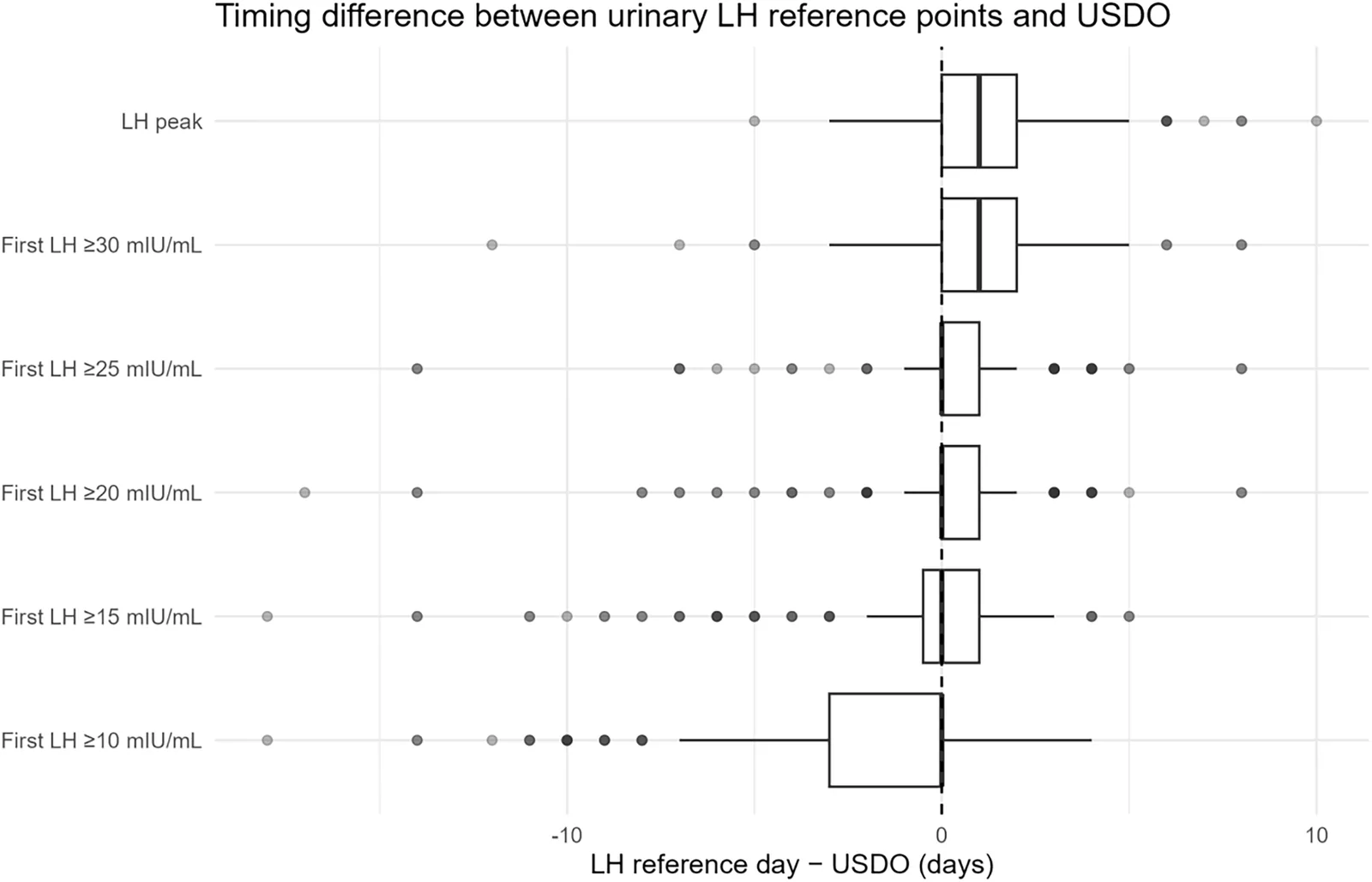
Timing difference between urinary luteinizing hormone (LH) reference points and ultrasound-determined ovulation (USDO). Boxplots show the distribution of the timing difference (LH reference day minus USDO) for the retrospectively identified LH peak and the first urinary LH concentrations reaching ≥10, ≥15, ≥20, ≥25, or ≥30 mIU/mL. The dashed vertical line at 0 represents USDO; negative values indicate that the LH reference event occurred before USDO, whereas positive values indicate that it occurred after USDO. Points beyond the whiskers represent individual outlying timing differences.

### Day-specific agreement between LH-guided and USDO-guided timing

In the unrestricted day-specific analysis, agreement varied by LH strategy and assessment day (**Supplementary Table S1**). For first urinary LH ≥15 mIU/mL, OPA was 75.3% (95% CI 69.9–81.2) at +6, 77.9% (73.5–82.8) at +7, 74.3% (67.9–78.7) at +8, and 82.2% (77.4–87.4) at +9. Corresponding AUC values were 0.764 (95% CI 0.698–0.826), 0.813 (0.756–0.871), 0.783 (0.715–0.840), and 0.835 (0.761–0.893), respectively.

Across LH thresholds, increasing the threshold generally improved temporal proximity to USDO but progressively reduced the number of evaluable cycles. Because the number of evaluable cycles differs among thresholds, the strategies are not always compared in identical samples, and apparent differences may reflect case composition as well as timing performance. No formal inferential comparison between LH thresholds was performed; the ranking of strategies is therefore descriptive rather than inferential, and ≥15 mIU/mL is best interpreted as a practical compromise between timing precision and cycle availability rather than as a statistically proven optimal threshold. These cluster-bootstrap intervals are wider than the confidence intervals obtained when cycles are treated as fully independent.

No single assessment day between +6 and +9 was uniformly superior across all LH strategies. The day-specific analysis therefore supports a practical mid-luteal assessment interval rather than a uniquely optimal fixed day.

### Sensitivity to the PdG classification threshold

Exploratory sensitivity analyses across PdG thresholds demonstrated that classification performance varied substantially with the selected cutoff (**Supplementary Table S1**). Lower PdG thresholds, particularly ≥5 µg/mL, produced very high positive percent agreement and positive predictive values but relatively poor negative predictive values, whereas ≥20 µg/mL produced high negative percent agreement and negative predictive values at the expense of markedly lower positive percent agreement. The ≥15 µg/mL threshold provided a comparatively balanced positive and negative agreement profile across LH strategies. Among prospective LH reference points, first urinary LH ≥20 mIU/mL showed slightly more balanced classification measures at PdG ≥15 µg/mL, but this modest numerical difference was accompanied by lower cycle availability than first LH ≥15 mIU/mL. Because no formal inferential comparison among LH thresholds was performed, first LH ≥15 mIU/mL was retained as a candidate practical reference on the basis of its temporal proximity to USDO and broader cycle availability rather than demonstrated statistical superiority. Within this strategy, day +9 showed the strongest descriptive performance at PdG ≥15 µg/mL (OPA 82.2%, PPA 79.4%, NPA 85.5%, PPV 86.2%, NPV 78.3%, AUC 0.835), although no formal inferential comparison between assessment days was performed (**Table 4**).

**Table 4:** Day-specific agreement between LH-timed and corresponding USDO-timed urinary PdG classification (threshold ≥15 µg/mL).

LH reference strategy	Day	Cycles (n)	OPA % (95% CI)	PPA % (95% CI)	NPA % (95% CI)	PPV % (95% CI)	NPV % (95% CI)	AUC (95% CI)	Women
LH peak	+6	279	73.1 (67.7–78.6)	79.5 (72.9–86.9)	67.8 (59.3–76.7)	67.3 (58.2–76.0)	79.8 (72.4–86.4)	0.773 (0.713–0.821)	102
LH peak	+7	277	71.5 (66.5–76.6)	69.3 (62.7–76.5)	74.0 (65.9–82.7)	75.9 (67.2–83.3)	67.1 (58.5–74.8)	0.772 (0.725–0.824)	102
LH peak	+8	275	73.1 (67.7–78.8)	69.1 (61.1–78.0)	77.0 (71.3–83.9)	74.6 (66.9–81.8)	71.8 (64.8–80.1)	0.789 (0.731–0.852)	101
LH peak	+9	269	76.6 (71.3–81.5)	72.5 (65.6–78.7)	80.9 (73.6–87.7)	80.0 (72.4–87.1)	73.6 (67.1–80.3)	0.816 (0.758–0.864)	101
First LH ≥10 mIU/mL	+6	266	75.6 (68.9–80.3)	65.0 (54.5–72.7)	84.6 (78.8–90.4)	78.4 (69.3–86.0)	73.8 (65.1–81.1)	0.714 (0.636–0.769)	99
First LH ≥10 mIU/mL	+7	264	70.5 (65.0–76.5)	57.9 (50.0–65.6)	85.7 (77.1–91.4)	83.2 (75.6–89.6)	62.6 (54.4–70.8)	0.693 (0.631–0.755)	99
First LH ≥10 mIU/mL	+8	265	77.7 (72.7–83.3)	72.6 (64.1–82.1)	83.1 (77.5–89.1)	81.7 (75.7–88.0)	74.5 (66.7–82.9)	0.811 (0.762–0.870)	98
First LH ≥10 mIU/mL	+9	261	77.4 (71.2–83.0)	69.4 (60.3–77.8)	85.8 (78.7–91.1)	83.8 (76.5–90.0)	72.7 (64.4–81.9)	0.782 (0.719–0.834)	98
First LH ≥15 mIU/mL	+6	243	75.3 (69.9–81.2)	74.8 (67.2–84.2)	75.8 (68.3–83.5)	73.5 (64.4–81.4)	77.0 (69.0–85.9)	0.764 (0.698–0.826)	96
First LH ≥15 mIU/mL	+7	240	77.9 (73.5–82.8)	74.1 (66.4–80.3)	82.9 (75.0–90.4)	84.7 (78.2–90.9)	71.3 (62.5–80.0)	0.813 (0.756–0.871)	95
First LH ≥15 mIU/mL	+8	241	74.3 (67.9–78.7)	71.3 (62.5–78.9)	77.7 (69.6–84.7)	78.6 (71.1–85.6)	70.2 (60.1–77.6)	0.783 (0.715–0.840)	95
First LH ≥15 mIU/mL	+9	236	82.2 (77.4–87.4)	79.4 (71.0–86.9)	85.5 (79.8–90.2)	86.2 (79.8–90.6)	78.3 (68.8–87.0)	0.835 (0.761–0.893)	96
First LH ≥20 mIU/mL	+6	215	76.3 (70.1–81.7)	77.9 (68.6–86.2)	74.8 (66.3–83.0)	74.3 (66.1–83.0)	78.3 (68.3–86.0)	0.790 (0.726–0.849)	93
First LH ≥20 mIU/mL	+7	212	78.3 (72.4–83.3)	79.1 (72.4–86.2)	77.3 (67.4–84.8)	80.5 (73.0–87.2)	75.8 (68.3–84.9)	0.818 (0.760–0.871)	92
First LH ≥20 mIU/mL	+8	213	77.0 (69.7–82.9)	75.9 (66.4–84.7)	78.2 (70.1–85.9)	79.4 (71.4–85.9)	74.5 (62.2–83.8)	0.804 (0.726–0.860)	92
First LH ≥20 mIU/mL	+9	208	78.8 (72.4–84.1)	76.3 (68.7–84.6)	81.9 (73.6–89.7)	83.7 (75.7–91.4)	74.0 (65.4–83.5)	0.809 (0.733–0.876)	93
First LH ≥25 mIU/mL	+6	191	72.8 (64.7–78.6)	77.5 (67.3–86.0)	68.6 (59.4–77.1)	68.3 (59.7–77.0)	77.8 (65.9–85.9)	0.761 (0.675–0.822)	88
First LH ≥25 mIU/mL	+7	188	77.7 (70.5–83.1)	77.2 (67.3–84.9)	78.2 (69.3–86.8)	80.4 (73.0–88.6)	74.7 (63.6–84.2)	0.809 (0.730–0.865)	86
First LH ≥25 mIU/mL	+8	187	74.3 (67.2–80.6)	72.2 (62.6–80.2)	76.7 (64.4–83.7)	76.9 (68.9–83.7)	71.9 (59.1–81.5)	0.782 (0.699–0.851)	87
First LH ≥25 mIU/mL	+9	185	78.4 (73.0–83.0)	74.0 (66.0–81.7)	83.5 (76.5–90.3)	84.1 (74.5–89.9)	73.2 (63.4–80.7)	0.824 (0.753–0.882)	88
First LH ≥30 mIU/mL	+6	163	73.6 (67.9–80.6)	83.1 (74.3–90.2)	65.1 (52.9–76.2)	68.1 (58.8–76.5)	81.2 (73.1–89.6)	0.798 (0.739–0.873)	82
First LH ≥30 mIU/mL	+7	161	78.9 (73.0–85.3)	80.4 (72.1–89.7)	76.8 (66.7–85.7)	82.2 (72.6–88.5)	74.6 (66.0–87.0)	0.812 (0.750–0.879)	81
First LH ≥30 mIU/mL	+8	161	74.5 (67.3–81.0)	77.1 (66.7–86.0)	71.8 (61.4–81.6)	74.4 (63.6–83.4)	74.7 (64.1–84.6)	0.773 (0.693–0.847)	82
First LH ≥30 mIU/mL	+9	161	79.5 (73.0–85.5)	75.5 (67.0–83.7)	85.1 (76.2–94.5)	87.7 (81.0–95.1)	71.2 (58.8–81.6)	0.861 (0.806–0.919)	83

For each assessment day, the LH-timed urinary PdG classification was compared with the PdG classification on the corresponding day after the ultrasound-determined day of ovulation (USDO), using a threshold of ≥15 µg/mL for both measurements. Values are point estimates with 95% confidence intervals estimated using participant-level cluster bootstrap to account for repeated cycles contributed by the same woman. OPA, overall percent agreement; PPA, positive percent agreement; NPA, negative percent agreement; PPV, positive predictive value; NPV, negative predictive value; AUC, area under the receiver operating characteristic curve. AUC was calculated using the continuous LH-timed PdG concentration against the binary corresponding-USDO-day PdG classification. LH concentrations are reported in mIU/mL and PdG concentrations in µg/mL.

## Discussion

### Principal findings

In this secondary analysis of ultrasound-confirmed ovulatory menstrual cycles, we evaluated LH-guided PdG timing. The day-specific analysis asked whether an LH-derived reference could reproduce the classification obtained on the corresponding fixed day after USDO.

For prospective use, first urinary LH ≥15 mIU/mL emerged as a candidate practical reference rather than a proven optimal threshold. It was centered on USDO, with approximately 70% of events within ±1 day and 81% within ±2 days, while retaining more evaluable cycles than higher thresholds. At the primary PdG threshold of ≥15 µg/mL, day-specific OPA for this strategy ranged from approximately 74% to 82%. Higher LH thresholds sometimes showed numerically higher agreement but were not consistently superior and reduced cycle availability. These findings should therefore be interpreted as hypothesis-generating and require prospective validation.

Past investigations have demonstrated that urinary PdG rises rapidly after ovulation and remains elevated throughout the mid-luteal phase, supporting its use as a non-invasive marker of luteal progesterone exposure (9, 14, 27). These studies have evaluated urinary PdG for ovulation confirmation or described normative hormone profiles relative to independently determined ovulation. However, relatively little attention has been directed toward identifying the most appropriate urinary LH-derived reference point for scheduling PdG assessment. Taken together, the findings support first urinary LH ≥15 mIU/mL as a practical prospective reference point rather than a uniquely optimal biological cutoff. The day-specific analysis supports its use for approximating ultrasound-referenced timing.

Based on this analysis, a urinary PdG concentration of ≥15 µg/mL was selected as the primary descriptive classification threshold, not as a diagnostic cutoff. Importantly, PdG ≥15 µg/mL should not be interpreted as defining “adequate” luteal function, nor should PdG <15 µg/mL be interpreted as defining inadequate luteal function. The threshold was additionally retained for three complementary reasons. First, the internal reference nomogram demonstrated that urinary PdG concentrations increase rapidly following ovulation and reach median values of approximately 15 µg/mL during the mid-luteal interval (USDO + 6 to +9), consistent with values reported for generally healthy ovulatory women (**Table 2** and **Figure 3**). Second, published studies report median urinary PdG concentrations of approximately 14–19 µg/mL during the same interval, placing 15 µg/mL near the expected mid-luteal range while acknowledging substantial biological variability. Third, a single descriptive cutoff permits transparent calculation of agreement measures and is technically feasible for future point-of-care or home-based quantitative assays. The sensitivity analysis did not identify a universally superior cutoff: lower thresholds emphasized positive agreement whereas higher thresholds emphasized negative agreement. Accordingly, ≥15 µg/mL was retained as a pragmatic, comparatively balanced methodological threshold. These data do not establish that a fixed threshold is preferable to an individualized or cycle-specific threshold; that question requires prospective validation linked to clinically meaningful outcomes.

The 15 µg/mL classification PdG threshold also differs from the ≥5 µg/mL criterion previously applied by our group for confirmation of ovulation on the basis of three consecutive positive measurements (4). The two values serve different purposes: the lower threshold identifies the post-ovulatory rise in PdG from follicular-phase baseline, whereas the present value is a mid-luteal classification marker positioned near the observed median of the sustained luteal plateau. Neither is proposed at the present as a diagnostic boundary for luteal phase deficiency.

### Clinical implications

The primary contribution of this study is methodological rather than diagnostic. The findings describe how closely an LH-timed urinary PdG classification reproduces the classification obtained on the corresponding day after USDO. They do not establish luteal sufficiency, implantation competence, fertility potential, or pregnancy prognosis. **Figure 5** summarizes the proposed prospective day-specific workflow.

**Figure 5:**
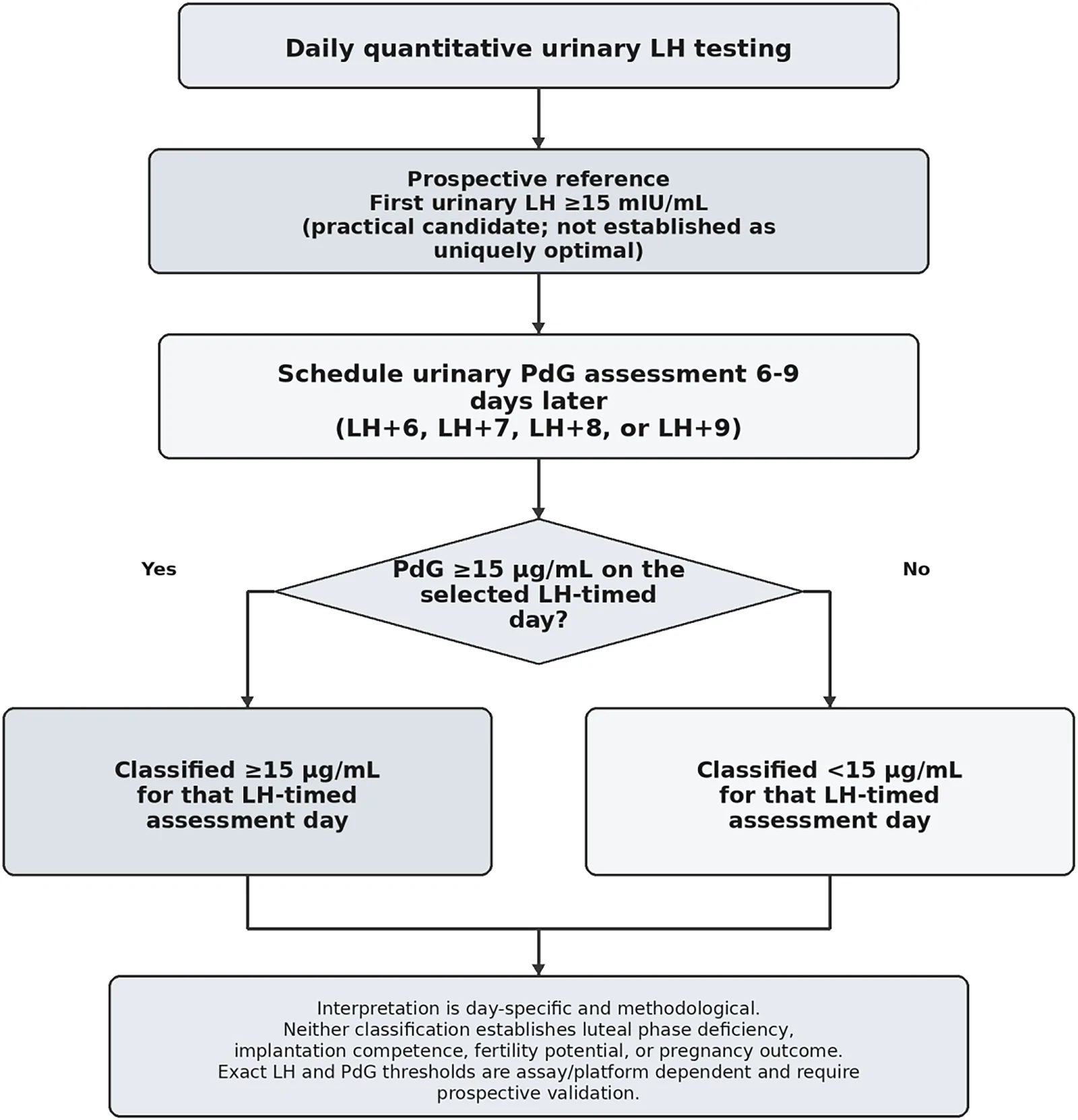
Proposed prospective timing framework for LH-guided day-specific urinary PdG assessment. First urinary LH ≥15 mIU/mL is shown as a practical candidate reference for scheduling PdG 6–9 days later, not as a uniquely optimal threshold. PdG ≥15 or <15 µg/mL represents a day-specific methodological classification, not a diagnosis of luteal phase deficiency, implantation competence, fertility potential, or pregnancy outcome. Research framework only — not a clinically validated decision rule. A below-threshold result does not define a diagnosis, treatment, or repeat-testing interval. Exact LH and PdG thresholds are assay- and platform-dependent and require prospective validation.

For prospective urinary hormone monitoring, first urinary LH ≥15 mIU/mL may be considered a candidate reference event for scheduling PdG assessment 6–9 days later. A PdG value above or below the selected threshold should be interpreted as a day-specific methodological classification for that LH-timed assessment day. A below-threshold value should not be interpreted as evidence of luteal insufficiency. The present analysis did not evaluate a serial repeat-testing strategy after a below-threshold result; therefore, no specific repeat-testing interval or clinical action can be recommended from these data. Accordingly, the present data do not establish a single LH-timed PdG measurement as clinically actionable. Neither a result above nor below the selected threshold should, on the basis of this study alone, determine a diagnosis, treatment decision, or repeat-testing schedule.

Urinary PdG is a downstream metabolite of progesterone and reflects progesterone exposure over the urine collection interval rather than a contemporaneous serum concentration. Although urinary and serum progesterone-related measures are biologically related, contemporaneous serum progesterone measurements were not available in this dataset. Accordingly, the urinary PdG threshold of 15 µg/mL cannot be assumed to correspond to a given serum progesterone concentration on the basis of the present data, and the LH-guided urinary timing strategy should not be extrapolated directly to serum progesterone testing without specific validation (9, 11).

The exact first-LH ≥15 mIU/mL strategy requires quantitative urinary LH measurement and cannot be assumed to transfer directly to qualitative ovulation predictor kits, cervical-mucus-only fertility awareness-based methods, or other nonquantitative approaches. The broader principle of using a cycle-specific physiological reference event may nevertheless be relevant to such methods, but requires separate validation. Home urinary monitoring may reduce patient burden and resource use compared with serial ultrasonography; however, no formal economic evaluation was undertaken, and cost-effectiveness cannot be concluded from this study.

### Strengths and limitations

Several strengths enhance the validity of this study. Ovulation was determined using serial transvaginal ultrasonography; participants underwent daily quantitative urinary hormone measurements; multiple prospectively applicable LH thresholds were evaluated within the same dataset; and this analysis avoided selecting cycles according to LH-USDO concordance. In addition, participant-level cluster bootstrap confidence intervals account for the repeated contribution of cycles by some women.

This study also has important limitations. It is a retrospective secondary analysis, and the LH timing strategies were not prospectively assigned. The number of evaluable cycles differed among LH thresholds because not all cycles reached the higher concentrations and because hormone measurements were occasionally missing. Participants were healthy women with ovulatory menstrual cycles; applicability to women with irregular cycles, ovulatory disorders, polycystic ovary syndrome, diminished ovarian reserve, infertility, hormonal contraception, or medical reproductive treatment cannot be assumed. The effects of participant age, body mass index, cycle length, and cycle regularity on LH-PdG agreement were not specifically evaluated and should not be inferred from these analyses.

The biological relationship between urinary LH timing and ovulation demonstrated here should be distinguished from the analytical transferability of the specific concentrations proposed. The underlying samples were collected in 1996–1997 and measured using laboratory-based time-resolved fluorometric immunoassays. Contemporary quantitative home urinary hormone devices employ different analytical principles, calibration systems, sample-handling procedures, and normalization conventions, and numerical thresholds are not automatically interchangeable across such systems. The transferable finding is the timing framework — scheduling PdG assessment approximately 6–9 days after a defined urinary LH reference event. The specific values of 15 mIU/mL for LH and 15 µg/mL for PdG are platform-dependent and would require prospective recalibration and validation on any modern device before being adopted. The analyses did not evaluate pregnancy outcomes, implantation, live birth, or other measures of reproductive success, and serum progesterone concentrations were not available for direct comparison. The PdG threshold of 15 µg/mL should therefore not be interpreted as a diagnostic cutoff for luteal phase deficiency, corpus luteum insufficiency, implantation competence, or reproductive prognosis unless it is future validated.

### Future directions

In recent years, there has been substantial growth in quantitative home urinary hormone monitors and smartphone- or AI-assisted urinary hormone tests. A recent prospective diagnostic accuracy study demonstrated that a quantitative home urinary hormone monitor could predict and confirm ovulation when compared with ultrasound (14). Future studies should prospectively validate the LH-guided timing strategies proposed here in independent populations and on contemporary monitoring platforms. Any AI-based approach intended to identify an LH peak prospectively should be evaluated as a locked algorithm that generates the reference event in real time, before the outcome is known, using a prespecified endpoint, comparison with USDO, and external validation in an independent cohort. Evaluation in women with irregular menstrual cycles, infertility, recurrent pregnancy loss, endocrine disorders, and medical reproductive treatment will be particularly important. Additional research should determine whether standardized LH-guided urinary PdG timing improves clinical decision-making, treatment monitoring, or reproductive outcomes. The present findings provide a hypothesis-generating methodological framework for such prospective validation studies.

## Conclusions

These hypothesis-generating findings suggest that urinary LH may provide a cycle-specific reference for scheduling day-specific mid-luteal urinary PdG assessment when serial ultrasonography is unavailable. this paper evaluates a timing methodology; it does not yet validate a clinical decision rule. First urinary LH ≥15 mIU/mL emerged as a candidate prospective reference because it was temporally close to USDO while retaining more evaluable cycles than higher thresholds. At PdG ≥15 µg/mL, day-specific agreement was moderate to high, but no LH threshold, PdG threshold, or assessment day was demonstrated to be uniquely optimal. A below-threshold result should be interpreted only as a day-specific methodological classification and not as evidence of luteal insufficiency. Prospective, platform-specific validation is required before clinical implementation.

## Data Availability

The datasets presented in this article are not readily available because specific data may be shared based on an approved data sharing agreement with original main researcher (please contact René Ecochard ecochardrene@gmail.com for further information). Requests to access the datasets should be directed to René Ecochard.
